# Supplementary figures and images for: Microbial Diversity and Community Structure of Wastewater-Driven Microalgal Biofilms
Source: Microorganisms. 2023 Dec 16;11(12):2994. doi: 10.3390/microorganisms11122994 (PMC10745310; doi:10.3390/microorganisms11122994)

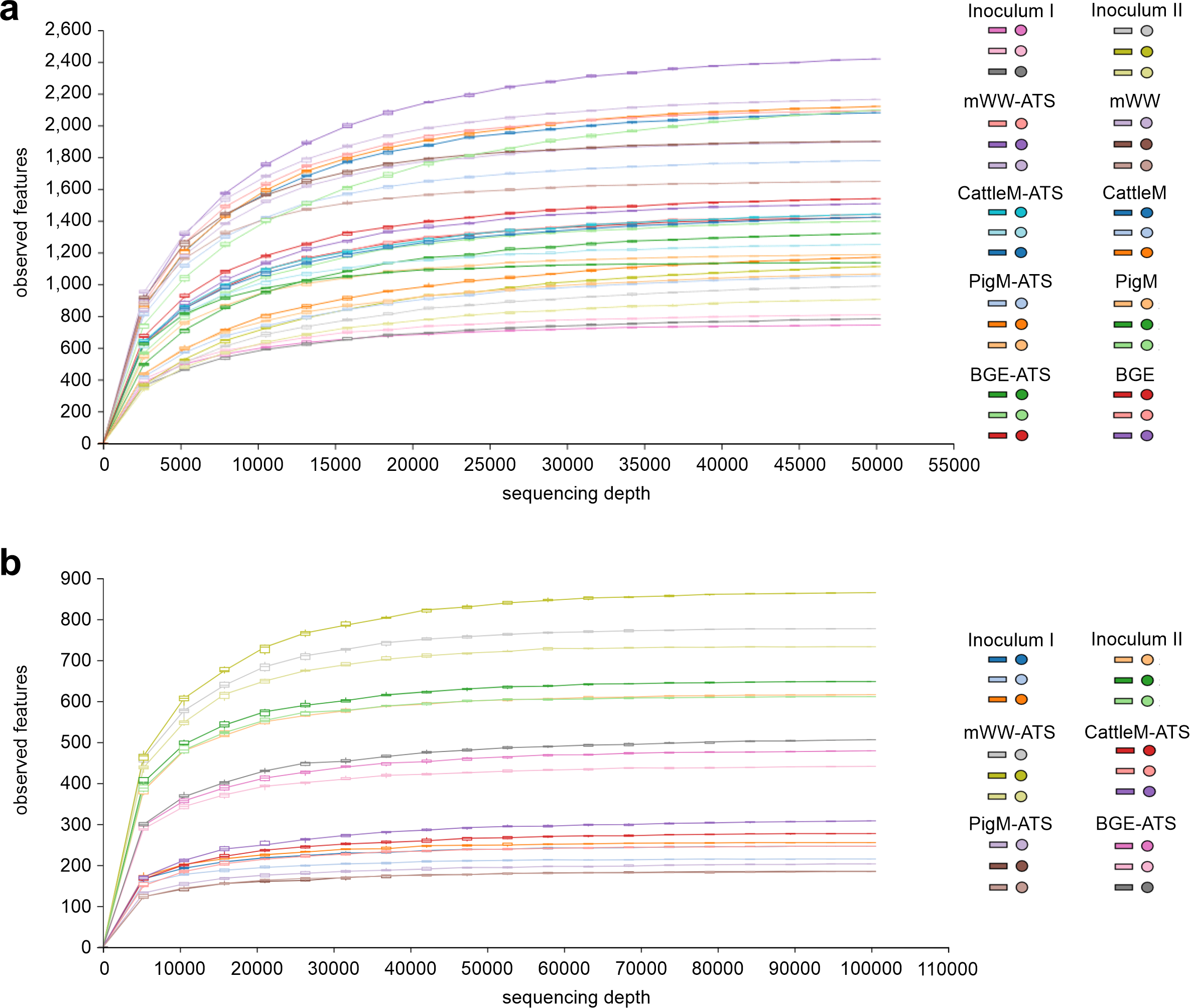

Supplement: Supplementary file 1 [file microorganisms-11-02994-s001.zip › FigS1.tif]

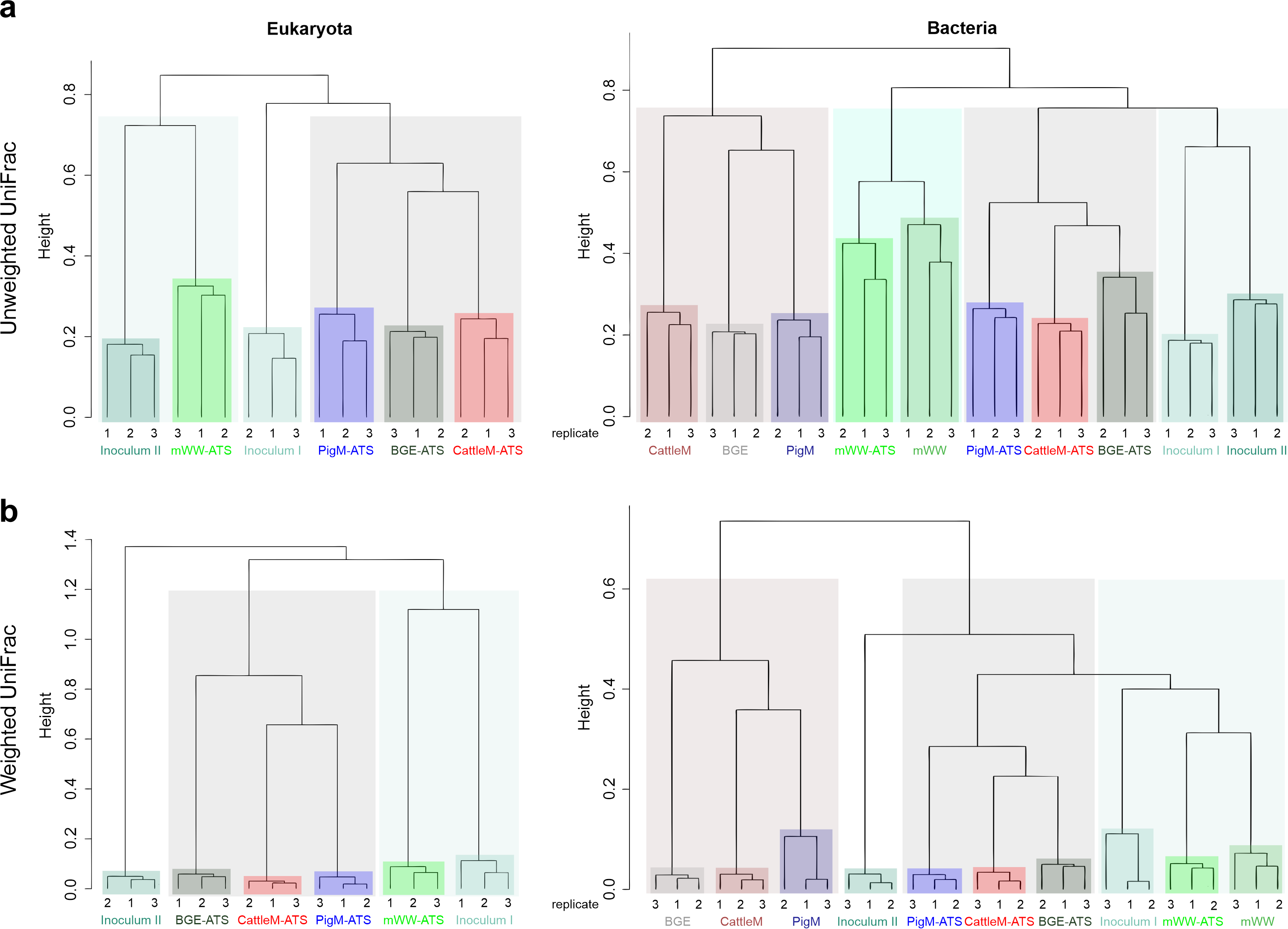

Supplement: Supplementary file 1 [file microorganisms-11-02994-s001.zip › FigS2.tif]
